# Muscle Health Before Sarcopenia: A Primary Care Approach to Preserving Strength, Function, and Healthy Longevity From Midlife

**DOI:** 10.7759/cureus.117480

**Published:** 2026-10-05

**Authors:** Marisleydis Perez Santiago, Raisa C Brana Miranda, Francisco J Santana Mercedes, Rosemarie Morera Gomez, Nathalie Rivero Rosabales

**Affiliations:** 1 Clinical Research, Primary Care - Research Initiative (PCRI), Miami Lakes, USA; 2 Internal Medicine, Hospital Menonita Ponce, Ponce, PRI; 3 Health, University of Louisville Physicians, Louisville, USA

**Keywords:** healthspan, healthy aging, longevity medicine, midlife, muscle health, primary care, resistance training, sarcopenia

## Abstract

Sarcopenia is traditionally framed as a geriatric condition characterized by age-related loss of muscle mass and strength. However, muscle mass, strength, power, and quality follow distinct age-related trajectories, with deterioration in several functional dimensions becoming detectable during midlife and, in some cases, preceding substantial changes in muscle quantity. A predominant focus on established sarcopenia may therefore overlook earlier opportunities to preserve muscle health. This narrative review examines the life-course trajectory of muscle health from midlife, evaluates evidence-based strategies for preserving muscle function, and considers practical opportunities for early identification in primary care. Resistance training is supported by substantial evidence for improving muscle strength, mass, power, and physical function, including evidence of benefit during midlife. Adequate dietary protein intake complements exercise-induced adaptations and may help preserve lean tissue, particularly during intentional weight loss, whereas routine vitamin D supplementation has not demonstrated comparable benefits for muscle outcomes in otherwise healthy adults. Current evidence remains insufficient to support universal muscle-health screening in asymptomatic middle-aged adults. Targeted, opportunistic case-finding may provide a pragmatic alternative in primary care, particularly for individuals with relevant comorbidities, functional concerns, nutritional risk, or periods of physical disuse. Brief assessment may incorporate objective measures such as handgrip strength and the Five Times Sit-to-Stand Test, whereas the relatively low sensitivity of SARC-F (Strength, Assistance with walking, Rise from a chair, Climb stairs, and Falls) may limit its utility for detecting subtle functional decline. Muscle-health deterioration is not confined to old age, and midlife represents a clinically relevant window for preserving strength, function, and muscular reserve. Current evidence supports resistance exercise, adequate nutrition, and physical activity as modifiable determinants of muscle health; however, prospective studies are needed to determine whether systematic midlife assessment and early intervention reduce future sarcopenia, disability, or loss of independence and improve long-term healthspan.

## Introduction and background

Skeletal muscle is increasingly recognized as an important determinant of health across the lifespan, with functions extending beyond locomotion to metabolism, bioenergetics, regeneration, interorgan communication, and physiological resilience [1]. Preserving muscle health may therefore be important not only for maintaining physical function but also for supporting overall health and quality of life throughout aging [1].

Sarcopenia represents a clinically important manifestation of impaired skeletal muscle health and is conceptually characterized by reduced muscle mass and muscle strength [2]. Its clinical consequences are substantial: sarcopenia is associated with reduced health-related quality of life and increased risks of falls, fractures, and mortality, with additional evidence linking sarcopenia to reduced instrumental activities of daily living [3]. However, a clinical focus centered primarily on established sarcopenia may identify muscle deterioration relatively late in its trajectory. Longitudinal and cross-sectional evidence across the adult lifespan indicates that changes in physical capacity and muscle strength can become detectable during midlife (defined in this review as 40-64 years), before substantial deterioration in some measures of muscle quantity occurs [4,5].

This earlier phase of the life course may represent an important opportunity for preserving muscle health. Muscle health remains responsive to modifiable behavioral factors, particularly resistance exercise, which can improve muscle strength, muscle size, muscle quality, and functional capacity in adults aged ≥50 years, although the supporting evidence extends into older age [6]. Primary care is particularly well positioned to address these factors through health promotion, risk assessment, and longitudinal care. Nevertheless, challenges remain in the identification and management of impaired muscle health in primary care, while much of the existing clinical framework remains focused on sarcopenia in older adults [7]. These limitations suggest an opportunity to broaden the clinical focus from identifying established sarcopenia toward preserving muscle health earlier in the life course.

A longevity medicine perspective shifts the emphasis from treating established disease and impairment toward preserving health and function across the lifespan, with the broader goal of extending healthspan [8]. Within this framework, muscle health may represent a modifiable component of healthy aging [1,6]. This review examines the trajectory of muscle health from midlife; the clinical relevance of muscle mass, strength, power, and quality; evidence-based strategies for preserving these dimensions; and practical opportunities for incorporating muscle health assessment and prevention into primary care. By focusing on muscle health before clinically overt sarcopenia, we aim to highlight a potentially important window for preserving physical function, independence, and healthspan.

## Review

Methods

Literature Search and Evidence Selection

This narrative review used a targeted approach to identify and synthesize literature on muscle health in midlife and its implications for primary care. PubMed, Embase, and Scopus were searched for English-language human studies published primarily from 2016 through September 2026; the last search was conducted on September 22, 2026. Search concepts included combinations of terms related to muscle health, sarcopenia, muscle strength and power, grip strength, midlife and aging, physical performance, resistance training and physical activity, protein intake, vitamin D, primary care assessment, and healthspan. Clinical guidelines and position statements from relevant professional societies were also reviewed directly. Reference lists of key articles and relevant guidelines were screened to identify additional sources. Priority was given to systematic reviews and meta-analyses, clinical guidelines and consensus or position statements, and longitudinal or large cohort studies. Narrative and scoping reviews and cross-sectional studies were included selectively when they addressed specific clinical questions. Earlier foundational or relevant studies were included when necessary to support established concepts, assessment methods, or foundational evidence. Study selection was guided by relevance to muscle-health trajectories, prevention, assessment, and practical application in primary care rather than by a formal systematic-review protocol.

From sarcopenia to lifelong muscle health

Sarcopenia has traditionally been framed primarily as a condition of older age, with contemporary definitions centered on reductions in muscle mass and muscle strength [2]. This framework has been clinically useful for identifying individuals with established impairment and increased risk of adverse outcomes [3]. However, a predominant focus on clinically manifest sarcopenia may inadvertently position muscle health as a concern that becomes relevant only after measurable deterioration has already occurred, potentially overlooking earlier opportunities to preserve muscle health.

Evidence across the adult lifespan suggests a broader trajectory of muscle deterioration. Muscle mass, strength, power, and quality follow distinct age-related patterns, with measurable deterioration in several dimensions becoming apparent during midlife (40-64 years) and, in some cases, preceding substantial changes in muscle quantity [4,5]. These findings support viewing age-related muscle deterioration as a life-course continuum rather than a process confined to older age. From this perspective, midlife may represent a clinically relevant period in which early changes in muscle health can be recognized while muscle function remains responsive to modifiable factors such as resistance exercise [6]. Reframing muscle health in this way shifts the clinical focus from identifying established sarcopenia alone toward recognizing earlier functional deterioration and potential opportunities for preserving muscle health in primary care. The following section examines this life-course trajectory across four interrelated dimensions of muscle health: mass, strength, power, and quality.

Muscle decline begins before old age

Age-related deterioration in skeletal muscle is increasingly understood as a gradual, life-course process rather than a phenomenon confined to older age, and changes in muscle function may become detectable from approximately 40 years of age, before substantial loss of muscle mass. A 47-year longitudinal study of a general population followed from adolescence into the seventh decade demonstrated that different components of physical capacity peaked between 19 and 36 years of age and subsequently declined, with the annual rate of loss accelerating from <1% in the first decade after peak performance to >2% in the final decade of observation [4]. A multinational pooling of eight Asian cohorts similarly found that handgrip strength begins to decline significantly in both sexes around 40 years, whereas surrogate measures of muscle quantity such as calf circumference deteriorate substantially later, typically after 60 years [5]. Together, these findings suggest that deterioration in muscle function may emerge during midlife while muscle quantity remains relatively preserved, identifying a clinically relevant window for recognizing early deterioration in muscle health before conventional thresholds for sarcopenia are reached.

Muscle Mass

Skeletal muscle is the body’s largest organ and the principal reservoir of body protein, accounting for roughly 30%-50% of body weight in men and 20%-40% in women [9]. Muscle mass begins a progressive decline from approximately 30 years of age, with estimated losses of approximately 3%-8% per decade [10]. Low muscle mass carries important prognostic value: a meta-analysis of 16 prospective cohort studies found that the lowest versus normal skeletal muscle mass index was associated with a 57% higher risk of all-cause mortality (risk ratio (RR), 1.57; 95% confidence interval (CI), 1.25-1.96) [9].

Muscle Strength

Muscle strength, most often quantified by handgrip dynamometry or the timed chair-stand test in ambulatory settings, may decline earlier and more substantially than muscle mass and is a central component of contemporary sarcopenia assessment frameworks [5,11]. Its prognostic value is broad and reproducible. In a systematic review and meta-analysis of 94 cohort studies, adults in the highest versus lowest handgrip category had substantially lower risks of incident cardiovascular disease (odds ratio (OR), 0.73), type 2 diabetes (OR, 0.79), musculoskeletal impairment (OR, 0.65), disability (OR, 0.57), depression (OR, 0.70), cognitive decline (OR, 0.57), and dementia (OR, 0.62); each 5-kg increment in grip strength was associated with a lower risk of most conditions, and better chair-stand performance showed parallel associations [12]. The overall certainty of evidence across outcomes ranged from very low to moderate [12].

These associations extend across multiple long-term health conditions, with several relationships also evident in the subgroup of adults aged <65 years analyzed in the meta-analysis [12]. Because these low-cost tests are feasible in primary care and track a decline that begins in midlife, strength may represent a particularly actionable marker of early deterioration in muscle health.

Muscle Power

Muscle power, the product of force and the velocity of its generation, captures the capacity for rapid force production that underlies rising from a chair, climbing stairs, and recovering from a stumble. Age-related differences in muscle power become apparent during midlife and may be proportionally greater than changes in muscle mass [13].

In the Copenhagen Sarcopenia Study, which included a cross-sectional cohort of 1,305 adults aged 20-93 years, relative lower-limb muscle power began to decline after approximately 40 years of age in both sexes, reflecting reductions in absolute and specific leg-extensor power together with increasing body mass [13].

Because muscle power shows age-related deterioration from midlife and underlies rapid everyday movements relevant to independence, it may represent a useful complementary dimension of functional muscle assessment [13].

Muscle Quality

Muscle quality refers to force or functional output per unit of muscle mass (neuromuscular quality) and to the composition of muscle tissue, particularly the infiltration of ectopic fat known as myosteatosis (morphological quality) [14]. The dissociation between quantity and quality is central to muscle aging, and therapies that increase mass without improving function are likely to be suboptimal. Myosteatosis is linked to insulin resistance and systemic inflammation and impairs contractile performance independent of, or synergistically with, low muscle mass [14].

Clinically, muscle quality may provide functional information beyond muscle quantity. In a large cohort, higher normal-attenuation muscle area was associated with better grip strength, gait speed, chair-stand, and walking performance across every tertile of muscle mass, independent of diabetes status [14]. These data reinforce that muscle quality may provide information about function beyond muscle quantity and that its assessment should not be conflated with measures of muscle size.

Long-Term Clinical Outcomes

The dimensions above converge on a consistent set of long-term, patient-important outcomes that define healthspan. The Global Leadership Initiative in Sarcopenia consensus concluded that sarcopenia is associated with reduced health-related quality of life and increased risks of mortality, falls, fractures, and reduced instrumental activities of daily living, although the strength of evidence varies across outcomes [3]. Reduced muscle strength, rather than low muscle mass alone, showed the most consistent association with mortality [3]. Because reduced muscle strength and impaired physical performance are associated with adverse outcomes, identifying deterioration in these dimensions before established sarcopenia may provide an opportunity to recognize individuals at increased functional risk using simple, inexpensive tools suited to primary care [3].

At the behavioral level, muscle-strengthening activity is associated with a 10%-17% lower risk of all-cause mortality, cardiovascular disease, total cancer, and diabetes, independent of aerobic activity and with maximal benefit at modest weekly volumes [15].

Two caveats temper this framing. First, the evidence base is predominantly observational, so muscle decline should be viewed both as a target for prevention and as a marker of broader biological aging, rather than assuming that increasing strength alone will prevent disease. Second, current evidence does not establish a benefit of universal population screening for muscle-health deterioration in asymptomatic middle-aged adults. A targeted case-finding approach may therefore represent a more pragmatic strategy while the clinical utility of systematic midlife screening remains uncertain. This reframes the clinical question from when sarcopenia is diagnosed to when meaningful deterioration in muscle health begins, positioning midlife strength, power, and quality as modifiable determinants of healthspan while substantial functional reserve remains. Longitudinal and interventional studies are needed to determine whether preserving or improving these dimensions in midlife modifies long-term clinical outcomes.

Preserving muscle health before sarcopenia

Because deterioration in muscle strength, power, and quality can become evident during midlife, including in adults in their 40s, prevention should not necessarily be deferred until sarcopenia is established [11,16]. The objective is not to treat sarcopenia prematurely, but to preserve muscular reserve while muscle-related functional outcomes remain responsive to physical activity interventions [17]. This is particularly relevant to primary care, where longitudinal encounters allow inadequate muscle loading, inactivity, nutritional risk, and periods of accelerated deconditioning to be addressed before functional impairment develops. Evidence for this approach, however, is uneven: resistance exercise and physical activity appear to improve muscle-related outcomes [17], but few trials have tested whether intervening in midlife specifically reduces later sarcopenia incidence. The rationale therefore rests on preserving modifiable determinants of muscle health, not on established prevention of a future diagnosis.

Resistance Training

Among available interventions, progressive resistance training (RT) is supported by some of the most direct evidence for improving muscle health. An umbrella review of 44 systematic reviews found that RT consistently increased skeletal muscle mass and strength compared with no exercise, with training load, volume, and frequency influencing some adaptations more than others [18]. A Bayesian network meta-analysis of 178 strength trials (n = 5,097) and 119 hypertrophy trials (n = 3,364) found that all RT prescriptions improved strength and hypertrophy compared with no exercise, with higher loads (≥80% one-repetition maximum (1RM)) most favorable for strength and multiple sets most favorable for hypertrophy [19]. The broad effectiveness across prescriptions suggests that initiation and sustained participation may matter more in preventive care than pursuing a theoretically optimal regimen.

The 2026 American College of Sports Medicine (ACSM) Position Stand further synthesizes this evidence base, drawing on 137 systematic reviews across more than 30,000 participants: RT improved strength, hypertrophy, power, endurance, contraction velocity, gait speed, balance, and physical function in healthy adults. Strength gains were enhanced by heavier loads (≥80% 1RM), full range of motion, two to three sets, and training at least twice weekly; hypertrophy by higher weekly volume; power by moderate loads moved rapidly during the concentric phase; and equipment type, complex periodization, and training to failure were not consistently necessary [20].

This power-specific finding deserves emphasis. Because muscle power, not maximal strength alone, appears to decline earlier and more steeply than mass with aging [13,16], it may be reasonable for RT prescriptions in midlife to include a velocity component (moving the concentric phase of a moderate-load repetition as quickly as safely possible) rather than relying exclusively on slow, maximal-strength-focused work. This remains a plausible extrapolation rather than a directly tested recommendation, since the evidence above comes from RT reviews in healthy adults generally, not from trials isolating midlife populations or comparing power-oriented against strength-only prescriptions. The approach is nonetheless well tolerated when technique is supervised, and progression is gradual, consistent with reviews identifying poor technique and excessive loading, not RT itself, as the main drivers of injury [21].

Evidence specific to midlife also supports intervening before old age: a meta-analysis of 66 studies in 3,387 adults aged 40-60 years found significant strength improvements after strength, combined strength-aerobic, strength-endurance, and water-based training, with strength and combined training also improving postural balance [17]. Heterogeneity and methodological quality were limiting factors, and no included studies evaluated falls, so long-term trials are still needed to confirm that these gains translate into prevention of later functional decline.

For primary care, this supports introducing RT as preventive behavior rather than waiting for clinically apparent weakness. Current guidelines recommend muscle-strengthening activity for all major muscle groups at least twice weekly, using machines, free weights, bands, or body weight [22]. For inexperienced or deconditioned patients, the priority is a feasible program that can be progressively advanced, not an immediately optimized prescription.

Physical Activity

Resistance exercise should be embedded within a broader active lifestyle, not treated as an isolated intervention. World Health Organization (WHO) guidelines recommend 150-300 minutes of moderate-intensity (or 75-150 minutes of vigorous-intensity) aerobic activity weekly, together with muscle-strengthening activity for major muscle groups at least twice weekly, and limiting sedentary time [22].

Aerobic activity and RT are not interchangeable for muscle preservation. Foundational exercise-prescription guidance treats cardiorespiratory training and resistance training as distinct stimuli, each targeting adaptations the other does not reliably produce [23]. In practice, aerobic exercise primarily supports cardiorespiratory and metabolic health, whereas progressive resistance exercise provides a more specific stimulus for strength and hypertrophy, while RT alone does not address the full spectrum of cardiometabolic benefit. A preventive strategy should therefore preserve both muscular and cardiorespiratory reserve rather than favor one at the expense of the other.

The midlife trial evidence described in the *Resistance Training* section extends here: the same 66-study meta-analysis found that strength-based and combined training improved balance as well as strength, confirming that functional outcomes remain modifiable during midlife itself, though, as noted, trial quality was modest [17].

Higher-intensity aerobic exercise may help when time is a barrier. A systematic review of eight controlled high-intensity interval training (HIIT) trials involving adults aged >55 years reported improvements in body composition in six of eight studies and improved strength in seven of eight studies, with improvements in gait speed and balance also reported [24]. Evidence was insufficient to establish superiority over moderate-intensity training, and because the included populations were older than 55 years, these findings should not be extrapolated uncritically across the full midlife age range defined in this review. HIIT is therefore best viewed as a time-efficient option for selected adults, not a preferred strategy.

These distinctions matter clinically: asking whether a patient "exercises" is insufficient. A patient may meet aerobic targets while performing little muscle-strengthening activity, or train with RT while remaining sedentary otherwise. Assessment should distinguish habitual movement, aerobic activity, muscle-strengthening activity, intensity, and sedentary time, and for patients citing lack of time, shorter, higher-intensity approaches may be considered, while moderate-intensity activity remains a well-supported default.

Periods of abrupt inactivity deserve particular attention. Muscle strength and mass can begin declining within days of bed rest, with the fastest rate of loss occurring early [25], and physical activity levels after hospitalization or acute illness do not reliably return to pre-event baseline even at follow-up [26]. Hospitalization, injury, acute illness, disease exacerbation, or major life transitions can therefore interrupt habitual activity rapidly and durably. These episodes are concentrated opportunities for primary care to flag the interruption and facilitate safe re-engagement, a preventive approach addressed further below.

Protein and Nutrition

Resistance exercise provides an important anabolic stimulus for maintaining or increasing skeletal muscle, while adaptation also depends on adequate energy and protein availability. Protein should therefore be considered complementary to muscle loading rather than an alternative to it [27].

A meta-analysis of 74 randomized controlled trials (RCTs) in healthy adults found that additional protein intake produced a small increase in lean body mass when combined with resistance exercise (standardized mean difference (SMD), 0.22; 95% CI, 0.14-0.30). Subgroup analyses suggested greater gains among participants aged <65 years in studies with total protein intakes ≥1.6 g/kg/day, whereas findings among adults aged ≥65 years in studies providing 1.2-1.59 g/kg/day were less certain and sensitive to individual studies [27].

A separate dose-response meta-analysis found that increased protein intake augmented strength gains when combined with RT, with a positive dose-response relationship up to approximately 1.5 g/kg/day and no further gains observed above that level [28]. Together, these findings argue against protein supplementation as an independent strategy: mechanical loading appears to be the primary driver of adaptation, and adequate protein availability supports, rather than substitutes for, that response. These intake ranges reflect training-related gains, not universal requirements for all middle-aged adults.

In primary care, the more relevant question is often whether energy and protein intake are adequate, not whether to prescribe a supplement. Unintentional weight loss, restrictive diets, reduced appetite, gastrointestinal disease, food insecurity, and caloric restriction can threaten lean tissue, and dietary assessment matters most when these coexist with inactivity.

Intentional weight loss deserves specific attention: loss of body weight can include fat-free mass as well as fat mass. A 2025 meta-analysis of 25 RCTs in adults with overweight or obesity found that adding resistance exercise to dietary weight loss attenuated fat-free mass loss (SMD, 0.40; moderate certainty), increased fat-mass loss (SMD, −0.36; high certainty), and improved strength (low certainty) [29]. Muscle preservation and obesity treatment need not be competing goals; the objective should extend beyond total weight loss to preserving functional lean tissue while preferentially reducing adiposity.

For most healthy middle-aged adults, a food-first strategy with adequate energy, protein, and diet quality is appropriate; supplementation should address an actual nutritional gap rather than substitute for diet or resistance exercise. A dedicated meta-analysis of protein-timing trials found that, after accounting for total protein intake, consuming protein in close temporal proximity to resistance exercise did not independently improve strength or hypertrophy; these findings suggest that total protein adequacy may be more important than precise peri-exercise timing [30].

Vitamin D

Vitamin D illustrates why a preventive framework must distinguish biological plausibility and observational association from evidence of intervention efficacy. Low circulating 25-hydroxyvitamin D (25(OH)D) has been associated with adverse musculoskeletal outcomes in observational studies, including a meta-analysis showing consistently lower 25(OH)D levels among adults with sarcopenia versus healthy controls [31], but this association does not establish that supplementation improves muscle health in otherwise healthy individuals.

A meta-analysis of 54 placebo-controlled RCTs (n = 8,747) found no overall benefit of vitamin D supplementation on muscle strength, mass, or performance; some outcomes modestly favored placebo [32]. The included populations were heterogeneous, with substantial representation of postmenopausal women and individuals with comorbidities rather than healthy adults specifically within the 40-64-year midlife range defined in this review; therefore, these findings argue against indiscriminate supplementation for muscle enhancement but should not be interpreted as evidence against treating an established deficiency.

This is consistent with the 2024 Endocrine Society guideline, which does not support routine empiric supplementation above dietary reference intakes for healthy adults under 75, nor routine 25-hydroxyvitamin D testing without an established indication [33]. Vitamin D should therefore occupy a supporting, not central, role: evaluation and treatment remain appropriate when risk factors or symptoms warrant them, but indiscriminate screening or high-dose supplementation is not supported as a substitute for resistance exercise, physical activity, or adequate nutrition.

Clinical Considerations and Safety

The role of primary care is not simply to advise patients to "exercise more and eat more protein." A preventive strategy requires identifying the circumstances that influence whether an individual can build, maintain, or lose muscular reserve, and tailoring intervention accordingly.

For most asymptomatic adults, activity can be initiated and progressed gradually without extensive workup. A consensus statement on long-term conditions concluded that the benefits of becoming more active substantially outweigh the risks, and that excessive risk messaging can itself be a barrier; routine preparticipation clearance is not supported for stable patients increasing activity gradually [34].

Resistance exercise itself should not be framed as intrinsically hazardous: a 2023 review of 28 studies found traditional strength training to have the lowest injury burden among resistance-training modalities, though methodological variability limited precise comparisons [35]. Safety is therefore better addressed through exercise selection, technique, and progressive loading than through routine restriction.

Evaluation before unrestricted progression is warranted for new exertional chest pain, syncope, unexplained severe dyspnea, acute illness, unstable cardiovascular disease, or significant new neurologic or musculoskeletal symptoms. Patients with substantial mobility impairment, balance problems, recent injury, or complex comorbidity may benefit from physical therapy, cardiac rehabilitation, or qualified exercise professionals rather than generic advice.

Nutritional advice likewise needs clinical context: intake levels tied to maximal training adaptations should not become blanket prescriptions. Patients with advanced kidney disease need individualized guidance, and unexplained weight loss, declining appetite, or loss of function should prompt evaluation for underlying disease rather than supplementation alone.

As already noted in the *Physical Activity* section, abrupt clinical transitions - inactivity, hospitalization, restrictive dieting, weight loss, worsening chronic disease - are concentrated opportunities to reassess activity, resistance exercise, and nutritional adequacy, not isolated events to manage only in passing.

Preserving muscle health before sarcopenia is therefore best conceptualized as longitudinal risk-factor management, not a discrete treatment: reinforcing resistance training and activity, identifying nutritional inadequacy, protecting lean tissue during weight loss, and facilitating recovery after inactivity, adjusted as health status changes. This avoids medicalizing normal aging while still treating skeletal muscle as a modifiable component of reserve.

The strength and limits of this evidence should remain explicit. RT has strong support for improving strength and mass, with increasingly direct midlife evidence for strength and balance; protein can augment adaptation to RT, and RT can protect fat-free mass during weight loss; routine vitamin D supplementation has not shown comparable benefit. What remains largely untested is the central longitudinal question: whether systematically preserving these determinants from midlife reduces incident sarcopenia, disability, or loss of independence decades later, which will require long-duration prospective studies beginning before old age.

Early identification in primary care

Primary care physicians are well positioned to recognize age-related deterioration in muscle health long before established sarcopenia develops, leveraging routine encounters and longitudinal follow-up from midlife (40-64 years) into older adulthood. Established frameworks, such as the European Working Group on Sarcopenia in Older People 2 (EWGSOP2) [11], the Sarcopenia Definition and Outcomes Consortium (SDOC) [36], and the Asian Working Group for Sarcopenia (AWGS) [37], have historically focused on defining, confirming, and categorizing established sarcopenia in geriatric populations.

This paradigm shift is underscored by the AWGS 2025 consensus update, which explicitly reframes sarcopenia management around a life-course approach to muscle health promotion. Three key refinements distinguish this framework: expanding diagnostic consideration to middle-aged adults (50-64 years) with age-specific thresholds, simplifying diagnostic criteria to concurrent low muscle mass and low muscle strength, with physical performance considered an outcome measure, and introducing a broader muscle-health framework that recognizes skeletal muscle as important for healthy longevity and emphasizes its cross-talk with other organ systems [37].

Despite these developments, current evidence does not establish a benefit of universal population screening for muscle-health deterioration in asymptomatic middle-aged adults, and validated early-intervention thresholds and cost-effectiveness data remain limited. A targeted, opportunistic case-finding approach may therefore represent a pragmatic strategy in primary care, particularly for individuals with relevant clinical risk factors or concerns, while the clinical utility of systematic midlife screening remains uncertain.

Target Populations for Case-Finding

Targeted assessment may be particularly relevant from midlife into older adulthood among individuals with clinical features, comorbidities, or recent health events associated with impaired muscle health or accelerated muscle loss. Primary-care data support the relevance of this approach: in a cross-sectional study of 213 adults aged ≥40 years, probable sarcopenia and sarcopenia were identified in 15.4% and 5.6% of participants, respectively, with diabetes, chronic kidney disease, and cardiovascular disease more common among those at risk [38].

Key clinical triggers for assessment may include chronic diseases associated with impaired muscle health, such as type 2 diabetes mellitus, chronic kidney disease, heart failure, and chronic obstructive pulmonary disease; unexplained symptoms or physical limitations, including persistent fatigue, subjective weakness, subtle mobility limitations, or recurrent falls; nutritional and weight-related concerns, such as unintentional weight loss, inadequate nutritional intake, or other clinical evidence of nutritional risk; and periods of accelerated deconditioning, including recent hospitalization, acute illness, prolonged bed rest, or other periods of substantially reduced physical activity. These features should be viewed as clinical prompts for targeted assessment rather than as validated indications for population screening.

Muscle Strength and Power Assessment

Muscle strength is a central component of contemporary sarcopenia assessment frameworks, including EWGSOP2, SDOC, and AWGS [11,36,37], and can be assessed objectively in clinical practice using simple functional measures [39]. Muscle power represents a complementary dimension of muscle function that may decline during midlife and may provide information beyond maximal strength, although it is not currently incorporated as a primary diagnostic criterion across these consensus frameworks.

Handgrip strength (HGS): Handgrip dynamometry is a rapid, objective, and portable measure of muscle strength. In a prospective UK Biobank study of 502,293 adults aged 40-69 years, lower handgrip strength was associated with higher risks of all-cause mortality and the incidence of and mortality from cardiovascular and respiratory diseases, as well as several cancer outcomes [40]. A systematic review of 48 prospective studies involving 3,135,473 participants found substantial heterogeneity in handgrip-strength measurement protocols, underscoring the importance of standardized assessment procedures [41]. International normative data from approximately 2.4 million adults across 69 countries and regions further demonstrate substantial age- and sex-related variation in handgrip strength, providing population-based context for interpretation across adulthood [42].

EWGSOP2 defines low handgrip strength as <27 kg in men and <16 kg in women [11]. For adults aged 50-64 years, AWGS 2025 introduced age-specific thresholds of <34 kg in men and <20 kg in women [37].

Five Times Sit-to-Stand Test (FTSST): The five-repetition chair-stand test provides a simple functional assessment of lower-extremity muscle performance using an armless chair and a stopwatch [43]. Under EWGSOP2, a completion time >15 seconds is used as an indicator of low muscle strength [11]. Age- and sex-specific reference values for the FTSST have also been established in a large population-based sample of 45,470 adults aged ≥50 years, providing normative context for interpretation across later adulthood [44]. In a longitudinal cohort of 2,943 adults aged 30-64 years followed for up to 18 years, inability to complete the Five Times Chair Stand Test (CST-5) was associated with more than twice the risk of all-cause mortality compared with completion in <13.7 seconds (hazard ratio (HR), 2.19; 95% CI, 1.65-2.92) [45]. Notably, half of participants who were unable to complete the test at the initial assessment reported no subjective difficulty rising from a chair [45].

Physical Performance and Functional Capacity

Usual gait speed: Gait speed provides an objective measure of physical performance that integrates multiple physiological systems involved in mobility. Normative data from 51,248 apparently healthy adults demonstrate age- and sex-related differences in usual gait speed across adulthood, providing useful context for interpretation [46]. Under EWGSOP2, a gait speed ≤0.8 m/s is considered low physical performance and is used to identify severe sarcopenia when low muscle strength and low muscle quantity or quality are also present [11]. Because this threshold was developed for sarcopenia assessment rather than for detecting early muscle-health deterioration in asymptomatic middle-aged adults, its role as an early-intervention threshold in midlife remains uncertain.

SARC-F questionnaire: SARC-F (Strength, Assistance with walking, Rise from a chair, Climb stairs, and Falls) is a five-item self-reported questionnaire used for sarcopenia case-finding, with a score ≥4 indicating increased risk and prompting further assessment [47]. In a diagnostic systematic review and meta-analysis including 35 studies and 19,707 participants, SARC-F demonstrated a pooled sensitivity of 36% (95% CI, 28-44%) and specificity of 87% (95% CI, 82-90%) [48]. Its relatively low sensitivity limits its ability to detect early or subtle muscle-health deterioration, particularly in asymptomatic middle-aged adults, for whom its performance has not been specifically established.

Muscle Quantity and Tissue Quality Assessment

Estimating muscle mass and quality complements functional testing.

Dual-energy X-ray absorptiometry (DXA): DXA provides estimates of appendicular lean mass (ALM) and is one of the methods recommended by EWGSOP2 for assessing low muscle quantity and confirming sarcopenia when low muscle strength has been identified [11]. Although widely used for body-composition assessment, DXA estimates lean soft tissue rather than contractile skeletal muscle directly, which should be considered when interpreting muscle quantity.

Bioelectrical impedance analysis (BIA): BIA provides a practical, portable method for estimating muscle quantity and is included in EWGSOP2 as an alternative to DXA for clinical assessment [11]. Because BIA-derived estimates rely on prediction equations rather than direct measurement of skeletal muscle, results should be interpreted using equations validated for the specific device and population. Hydration status and other factors affecting body-fluid distribution may also influence these estimates.

Muscle quality (myosteatosis): Computed tomography (CT)-derived measures can characterize fat infiltration within skeletal muscle. These measures have been associated with handgrip strength and physical performance independently of muscle mass and diabetes status [14], supporting muscle quality as a complementary dimension of muscle-health assessment.

A Pragmatic Four-Step Workflow for Primary Care

To address implementation barriers such as limited consultation time and access to specialized equipment, we propose a pragmatic, hypothesis-generating four-step case-finding framework adapted from the approach described by Daly et al. [49]. This framework is intended to support opportunistic clinical assessment in primary care and should not be interpreted as a validated screening algorithm or guideline-endorsed pathway. Its clinical utility and the appropriate thresholds for intervention require prospective validation. The sequence of case-finding steps, the suggested reassessment frequency, and the clinical response to subthreshold abnormalities should be regarded as expert-informed, pragmatic extrapolations rather than prospectively validated strategies for preventing sarcopenia in asymptomatic middle-aged adults. In contrast, the lifestyle interventions incorporated into the framework are supported by established evidence and, where applicable, current physical-activity guidance, particularly progressive resistance exercise [18-20,22] and adequate protein intake tailored to nutritional status, activity level, and clinical context [27,28].

Suspect: Identify individuals with clinical risk factors, symptoms, comorbidities, or recent health events associated with poor muscle health or accelerated muscle loss. This risk-based starting point is an expert-informed approach.

Ask and assess: Use objective measures of muscle function, including handgrip strength [39-42] and the FTSST [43-45], with brief case-finding tools such as SARC-F [47,48] as a complement, given its limited sensitivity. Gait speed may provide additional information on physical performance [39,46], and, where clinically indicated and available, DXA or BIA may be used to assess muscle quantity [11]. These additional measures broaden the clinical assessment beyond the core case-finding sequence summarized here. The sequence itself represents an expert-informed case-finding approach rather than a prospectively validated pathway.

Prevent or manage: For individuals with clinical risk factors or reduced muscle-function measures, consider reinforcing evidence-based lifestyle measures, particularly progressive resistance exercise at least twice weekly [18-20,22] and adequate dietary protein intake tailored to nutritional status, activity level, and clinical context [27,28]. Published cut points, including AWGS 2025 thresholds for adults aged 50-64 years [37], may provide diagnostic context but were developed to identify sarcopenia rather than to define validated early-intervention thresholds. Accordingly, using subthreshold findings to trigger intervention is a pragmatic clinical extrapolation, whereas the lifestyle measures themselves are evidence-based.

Review: Consider reassessing muscle health annually or after major health events, such as falls, fractures, immobilization, or hospitalization, and when clinically relevant changes in health status or risk factors occur [49]. This suggested follow-up interval is adapted from the proposed framework of Daly et al. [49] and represents an expert-informed approach; it has not been prospectively validated as an optimal reassessment schedule for asymptomatic middle-aged adults.

Practical muscle-health assessment tools for primary care and their key limitations in midlife are summarized in Table 1.

**Table 1:** Summary of primary care muscle-health assessment tools. * Approximate administration times are pragmatic estimates for routine clinical workflow rather than validated comparative timing benchmarks. HGS, handgrip strength; FTSST, Five Times Sit-to-Stand Test; DXA, dual-energy X-ray absorptiometry; BIA, bioelectrical impedance analysis; SARC-F, Strength, Assistance with walking, Rise from a chair, Climb stairs, and Falls.

Measure	Domain	Time*	Equipment	Established role	Key midlife limitation
HGS	Strength	~2-3 min	Dynamometer	Assessment of low muscle strength [39-42]	No validated early-intervention threshold
FTSST	Lower-extremity muscle performance	<2 min	Chair + timer	Lower-extremity strength assessment [43-45]	Multifactorial performance; threshold uncertainty
Gait speed	Integrated physical performance	~2-3 min	Walkway + timer	Physical performance assessment [39,46]	No validated early-intervention threshold for otherwise healthy midlife adults
SARC-F	Self-reported function	<2 min	None	Case-finding [47,48]	Limited sensitivity; limited evidence for asymptomatic midlife decline
DXA	Muscle quantity/body composition	~10-20 min	DXA scanner	Assessment of muscle quantity [11]	Access/cost; muscle quantity does not directly measure contractile function
BIA	Estimated muscle quantity/body composition	~2-5 min	BIA device	Alternative assessment of muscle quantity [11]	Role in routine midlife muscle-health assessment remains uncertain

Muscle health and longevity medicine

Longevity medicine emphasizes extending healthspan alongside lifespan, with a focus on preserving health and function across aging [8]. Skeletal muscle is relevant to this goal because muscle health depends on interconnected processes involving metabolism and bioenergetics, regeneration, structural integrity, and interorgan cross-talk that collectively support adaptability and physiological resilience [1]. Strength, power, and physical capacity show age-related changes across the adult life course [4,5,13]. Lower muscular fitness has also been associated with adverse long-term health outcomes, including disability, cardiometabolic disease, and cognitive outcomes [12], while lower handgrip strength has been associated with all-cause mortality [40].

A life-course perspective therefore shifts the clinical question from whether sarcopenia is already present to whether an unfavorable functional trajectory can be recognized while substantial reserve remains. This should not redefine normal aging as disease. Rather, muscle health can be viewed as a modifiable component of healthy aging, supported by evidence that resistance training can improve multiple muscle-related outcomes, including strength, muscle size, muscle quality, and functional capacity, in adults aged ≥50 years, although the supporting evidence extends into older age [6].

The distinction between biological plausibility and proven clinical utility is essential. Associations between muscular fitness and mortality do not demonstrate that measuring HGS, gait speed, or chair-stand performance extends healthspan, and evidence that exercise improves strength does not establish that a particular screening program reduces future disability. At present, a key rationale for incorporating muscle health into preventive care is functional: preserving the capacity required for mobility, independence, metabolic health, and resilience. A longevity-oriented approach should therefore complement, rather than replace, established sarcopenia frameworks while prospective evidence on earlier assessment continues to develop.

Evidence gaps and future directions

First, the optimal age and target population for assessment are unknown, including when within the midlife range defined in this review (40-64 years) assessment should begin. Second, the preferred strategy (HGS, FTSST, gait speed, body composition, or a multidimensional approach) has not been established. Third, established sarcopenia cut points should not automatically be interpreted as early-intervention thresholds in otherwise healthy middle-aged adults. Although AWGS 2025 introduced age-specific thresholds for adults aged 50-64 years [37], these diagnostic thresholds do not establish when preventive intervention should begin. Age- and sex-specific normative values can provide clinical context but should not be interpreted as intervention thresholds. Longitudinal assessment of within-person changes may offer additional information beyond a single measurement, but the clinical significance and optimal magnitude of change require prospective validation. Similarly, the case-finding sequence, reassessment interval, and response to subthreshold findings proposed in the *Pragmatic Four-Step Workflow for Primary Care* section require prospective validation.

Fourth, clinical utility must be demonstrated. Pragmatic or randomized trials should test whether identifying reduced or declining muscle function in midlife and linking these findings to targeted interventions improves strength, physical performance, disability-free survival, or other healthspan-related outcomes compared with usual care.

Future studies should evaluate cost-effectiveness, patient acceptability, implementation burden, potential harms of labeling, and measurement performance across sex, body size, race and ethnicity, socioeconomic conditions, and comorbidity. Standardized measurement will also be important if muscle-health trajectories are to be integrated into electronic health records or compared across health systems.

Current evidence supports skeletal muscle as an important contributor to physical function across adulthood but remains insufficient to establish the clinical benefit of universal muscle-health screening in asymptomatic middle-aged adults. The next step is not simply earlier diagnosis of sarcopenia, but the development and testing of a life-course framework that can distinguish normal age-related change from clinically meaningful early deterioration and determine whether assessment linked to targeted intervention preserves function, independence, and long-term healthspan.

Limitations

This review has several limitations. As a narrative review, it did not follow a formal systematic-review protocol, was not prospectively registered, and did not include a structured assessment of risk of bias or certainty of evidence; study selection therefore involved judgment and may be subject to selection bias. The search was restricted to English-language human studies and to the databases and guideline sources described in the Methods, so relevant studies published in other languages or indexed elsewhere may have been missed. Much of the supporting evidence derives from observational cohorts, older adults, or populations that only partly overlap with the midlife range defined here (40-64 years), and the proposed four-step workflow and the interpretation of subthreshold findings are expert-informed rather than prospectively validated. Finally, this is a rapidly evolving field, and studies published after the last search are not captured.

## Conclusions

Muscle health deterioration can become detectable during midlife, with declines in strength, power, and quality in some cases preceding substantial changes in muscle quantity. Primary care is well positioned to shift the clinical focus from recognition of established sarcopenia alone toward earlier preservation of muscular reserve and physical function across the life course.

Although current evidence does not establish the clinical benefit of universal muscle-health screening in asymptomatic middle-aged adults, targeted opportunistic case-finding using accessible functional measures such as handgrip strength and the FTSST may provide a pragmatic approach to clinical assessment in individuals with relevant risk factors or clinical concerns, rather than a validated screening strategy. Substantial evidence supports progressive resistance training, together with adequate nutrition and protein intake, for preserving or improving muscle-related outcomes, whereas routine vitamin D supplementation has not demonstrated comparable benefit for muscle health in otherwise healthy adults. Midlife therefore represents a potentially important window for preserving muscle health and supporting healthy longevity, although prospective studies are needed to determine whether earlier assessment and intervention translate into reduced disability, incident sarcopenia, or improved long-term healthspan.
